# Supplementary material for: Extensive fragmentation and re-organization of transcription in Systemic Lupus Erythematosus
Source: Sci Rep. 2020 Oct 6;10:16648. doi: 10.1038/s41598-020-73654-4 (PMC7539002; doi:10.1038/s41598-020-73654-4)
Supplement: Supplementary file 1 — Supplementary Figures. [file 41598_2020_73654_MOESM1_ESM.docx]

**Supplementary Information**

**Extensive fragmentation and re-organization of transcription in Systemic Lupus Erythematosus**

Vasilis F. Ntasis^1^, Nikolaos I. Panousis^2-5^, Maria G. Tektonidou^6,7^, Emmanouil T. Dermitzakis^2-4,8^, Dimitrios T. Boumpas^7-10^, George K. Bertsias^11,12^, Christoforos Nikolaou^1,12,13^*

^1^Department of Biology, University of Crete, Heraklion, 70013, Greece

^2^Department of Genetic Medicine and Development, University of Geneva Medical School, Geneva, Switzerland

^3^Institute of Genetics and Genomics in Geneva (iG3), University of Geneva Medical School, Geneva, Switzerland;

^4^Swiss Institute of Bioinformatics, Geneva, Switzerland;

^5^Wellcome Sanger Institute, Hinxton, United Kingdom

^6^Department of Propaedeutic Internal Medicine, Medical School, National and Kapodistrian University of Athens, Greece

^7^Joint Academic Rheumatology Program, Medical School, National and Kapodistrian University of Athens, Greece

^8^Biomedical Research Foundation of the Academy of Athens, Athens, Greece.

^9^4th Department of Medicine, Attikon University Hospital, National and Kapodistrian University of Athens Medical School, Athens, Greece

^10^Medical school, University of Cyprus, Nicosia, Cyprus

^11^Department of Rheumatology, Clinical Immunology, Medical School, University of Crete, Heraklion, 70013, Greece

^12^Institute of Molecular Biology and Biotechnology (IMBB), Foundation of Research and Technology (FORTH), Heraklion, Greece

^13^Institute of Bioinnovation, Biomedical Sciences Research Center “Alexander Fleming”, Athens, Greece

*Corresponding Author:

email: [cnikolaou@fleming.gr](mailto:cnikolaou@fleming.gr)

**RESULTS**

**DCE patterns are not related to sequencing output, genomic distribution of reads or cell- type heterogeneity**

One observation that particularly stands out in the co-expression patterns between SLE patient and healthy individual genomes, is the increased fragmentation in the samples of low-activity patients. This is somewhat contrasting to the increased overall gene deregulation, occurring in high-activity patients and, more importantly, suggests that disjointed gene regulation is characteristic of the genomes of patients with less pronounced molecular and pathological manifestations. In order to assess possible methodological biases underlying this key observation, we compared the three disease activity groups, analyzed in this study (low, intermediate and high) with the healthy individuals at various levels, including sequencing output, genomic distribution of reds and cell-type heterogeneity.

We compared total read counts and mapped read counts for each sample according to the disease group in **Supplementary Figure 1**. No significant changes were observed, besides a small heterogeneity within the high-activity group, likely due to the smaller sample size (26 patients compared to 55 and 61 for low- and intermediate activity respectively).


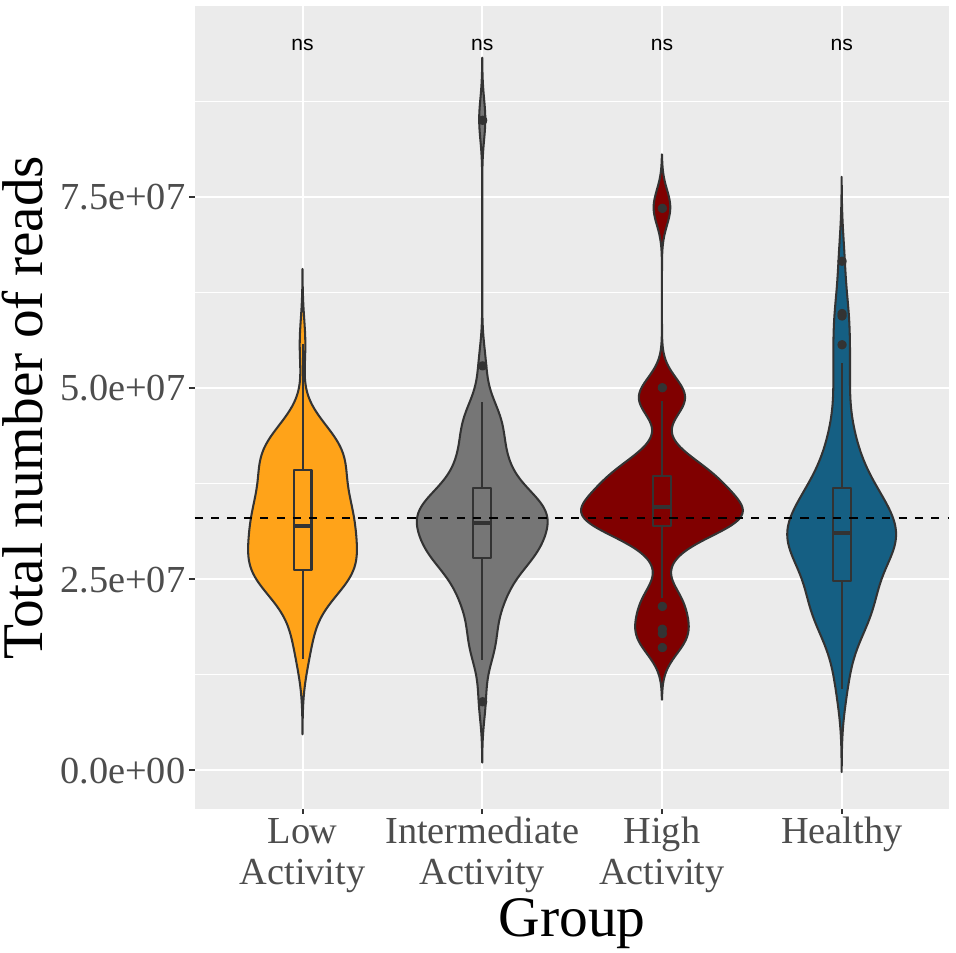

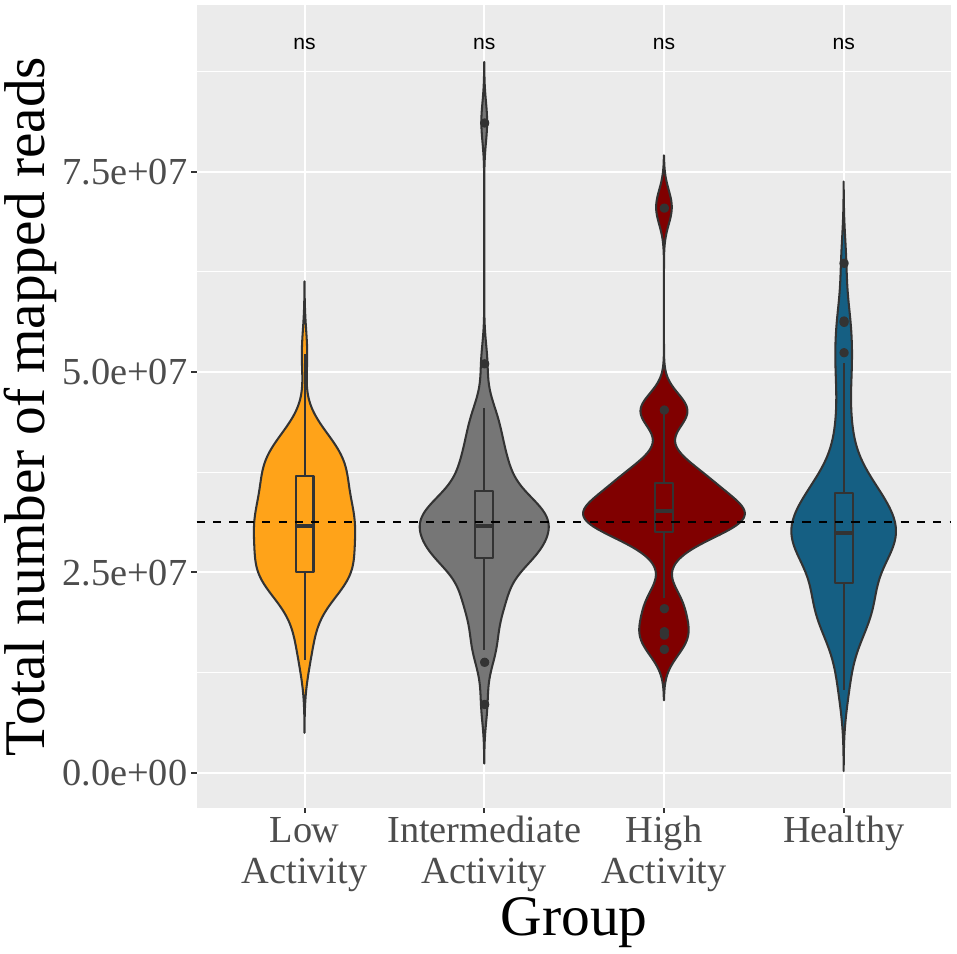


**Supplementary Figure 1**. Total reads (left) and total mapped reads (right) of genomes belonging to the different groups studied. The results of Mann-Whitney-Wilcoxon tests comparing each group to the global read count distribution, whose average value is indicated by a dashed line, are demonstrated by the significance level indicators. No significant differences were observed.

More importantly, low-activity genomes have read counts which are very similar to those of healthy individuals, even though they show the most strikingly different co-expression patterns, which is highly suggestive of the latter being independent of sequencing output.

Similar read counts may be distributed unevenly in annotated regions of the genome during the mapping process and localized biases at this point may bring about variability in the observed co-expression patterns. In order to test for this we conducted a Principal Component Analysis using the normalized gene counts for all 27061 genes used in our analysis. The results, shown in Supplementary Figure 2, are characteristically invariant, meaning that reads were distributed evenly in the annotated proportion of the genome, independently of disease activity group.


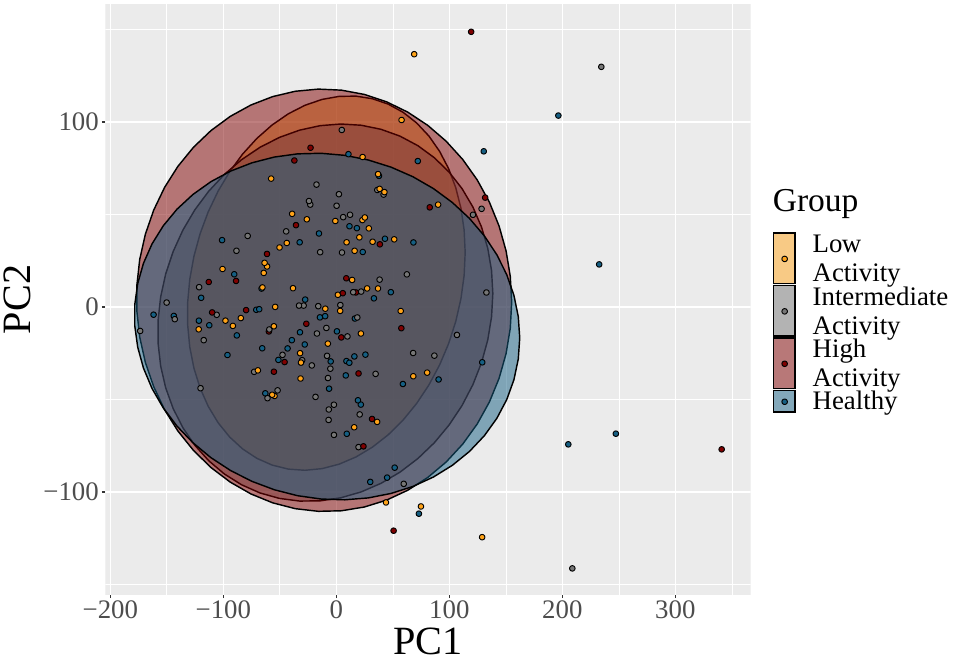


**Supplementary Figure 2**. Scatterplot illustrating the first two principal components resulted by a PCA for the 200 samples analyzed in the study. Normalized gene counts for 27061 genes were used as initial features. Each coloured point represents a sample. Disease activity groups and healthy individuals are grouped in coloured ellipses.

A last possible source of bias may stem from the fact that our approach is based on gene expression from a heterogeneous cell population. In order to assess the extent, to which the differences in the DCE patterns and the more fragmented distribution in SLE patients may be attributed to cell population heterogeneity we used the inferred cell type distribution acquired for the same dataset^1^. We actually found healthy samples to be more heterogeneous in terms of different cell type representations (**Supplementary Figure 3**). That is, the more fragmented patterns stem from the less homogeneous cell samples and therefore cannot be attributed to cell-type expression variability.


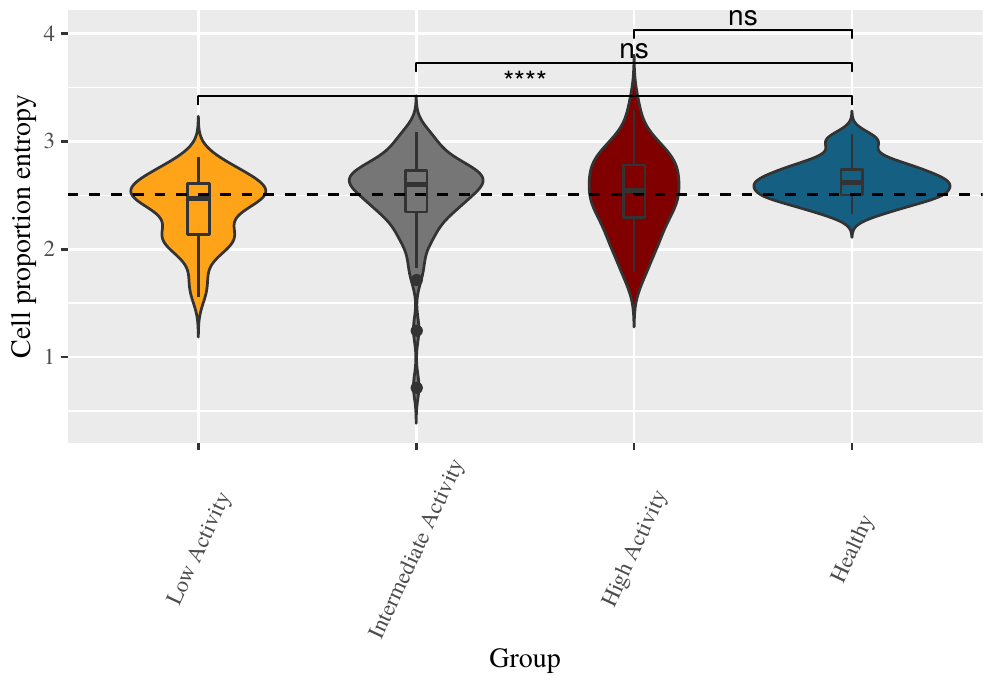


**Supplementary Figure 3. Cell proportion distribution.** Violin plots presenting the estimated distribution of cell proportion entropy (see **Methods, Main Paper**) per group studied. The results of Mann-Whitney-Wilcoxon tests comparing each patient group to the healthy group are demonstrated by the significance level indicators. Classic boxplots are included. Dashed line represents the overall average value of the depicted variable.

**BP-Score comparison**

A simple value measure of DCE pattern similarity may be obtained with an assessment of genomic coordinate changes through the implementation of BPscore^2^, which allowed us to see that even if the high-activity DCEs are comparable in terms of genome coverage with the healthy ones, they were radically different in terms of coordinates as suggested by the BP scores (**Supplementary Figure 4**). High BP scores are also observed when comparing high with low activity genomes (data not shown) a fact that is strongly suggestive of a general re-distribution of co-expression in different domains, accompanying the more extensive gene expression changes in high activity patients.

*
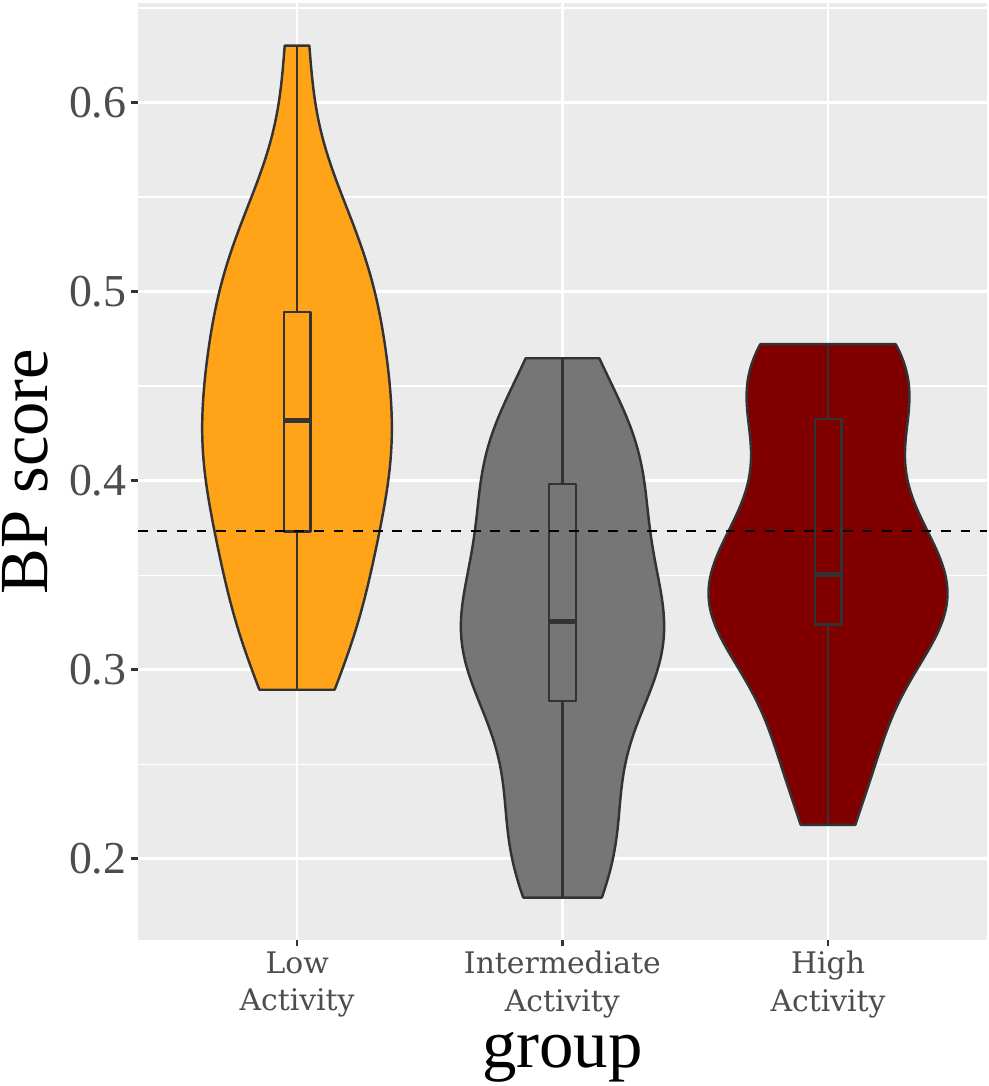
*

**Supplementary Figure 4. BP distance score characterizing the different patient groups.** Violin plots presenting the estimated distribution of BP distance score (per chromosome) between the DCE profiles of patient groups and the healthy control group. Classic boxplots are included. Dashed line represents the overall mean BP score value.

**Modular Anaysis of Differential Gene Expression and Weighted Gene Co-expression Network Analysis (WGCNA) identify gene sets that are associated with distinct clinical manifestations of SLE**

We used a recently published gene expression dataset on SLE^1^⁠ to assess the differential expression levels (against healthy individuals) for three distinct patient groups according to disease activity based on the SLEDAI index (see **Methods, Main Paper**). We employed a modular analysis of differential gene expression (**Methods, Main Paper**) which uncovered quantitative differences in key pathways across disease groups. Functional modules strongly associated with SLE, such as interferon signaling, neutrophil activation and the innate antiviral response, showed gradually increasing enrichments from low to high activity state (**Supplementary Figure 5**). More general biological functions such as cell cycle, primarily associated with T-cell division, become enriched only in high activity patients. Functions pertaining to plasma cells and B-cells were enriched in under-expressed genes, yet this enrichment was inversely associated with disease activity (**Supplementary Figure 5**. These results are suggestive of quantifiable phenotypic variability between patients with different clinical activity states, in agreement with the previously defined susceptibility and severity gene signatures^1^.

We took advantage of the detailed clinical information (including hematological and immunological data and macroscopic observations) that was available, by combining it with a *Weighted Gene Co-expression Network Analysis* (WGCNA, see **Methods, Main Paper**). We identified significant co-expression modules, that is, groups of genes that tend to have similar expression levels across healthy individuals and SLE patients. These modules were then associated with available clinical traits at single-patient level, in order to define gene sub-signatures of clinical relevance. A number of gene modules were highly correlated with disease activity (SLEDAI), while distinct gene sets correlated with clinical traits such as active nephritis, biomarkers such as anti-double stranded DNA antibodies, cell-specific attributes for neutrophil, plasma and B-cells, as well as molecular pathways such as the interferon signaling (**Supplementary Figure 6**).


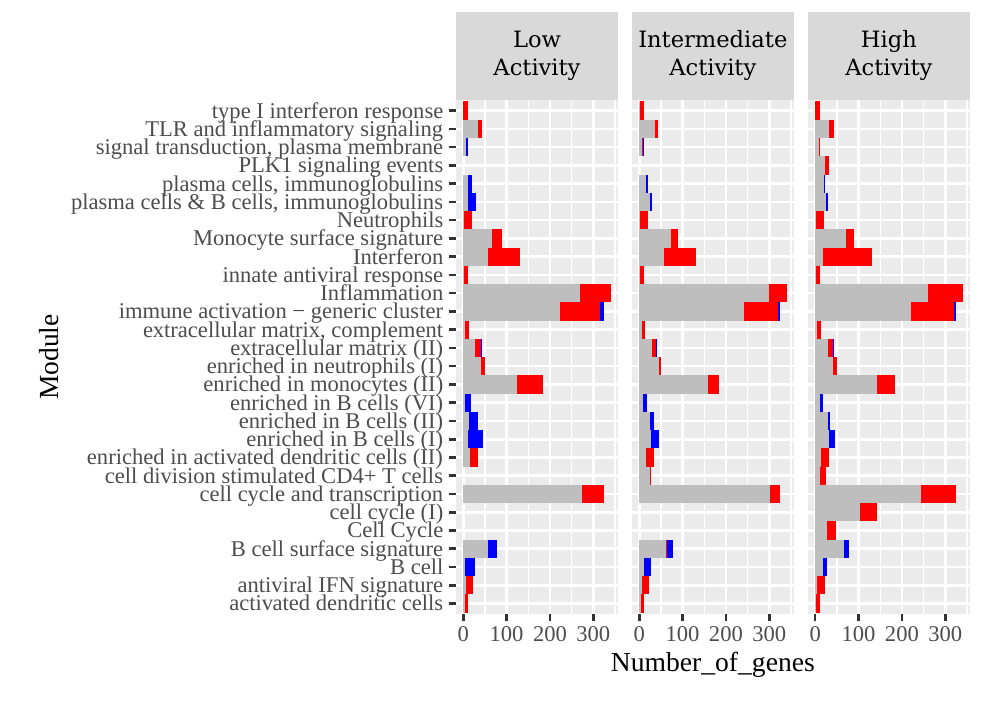


**Supplementary Figure 5**. **Gene set enrichment analysis of differential patient expression.** Barplots illustrating the results of gene set enrichment for blood modules (see **Methods, Main Paper**). Genes expressed in each patient group were sorted according to their log-FoldChange (calculated in regard to the control healthy group) for this analysis. Top significant modules for each patient group, based on the CERNO test (corrected p-value <= 0.05)^3^⁠are presented. The overall length of each bar illustrates the total number of genes, which are members of the respective module. Overexpressed DEGs are represented by the red part on each bar. Underexpressed DEGs are represented by the blue part on each bar.

**
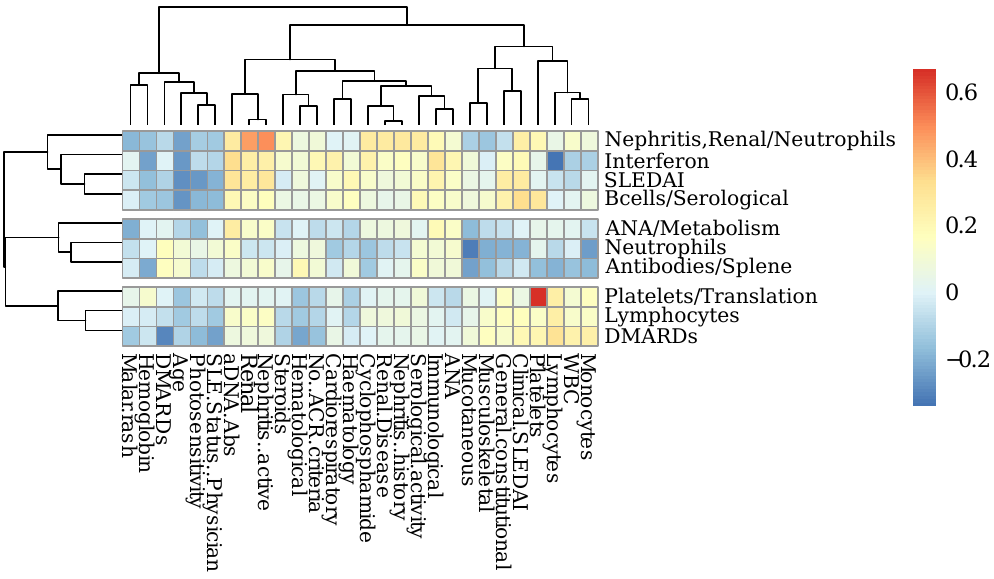
**

**Supplementary Figure 6. Correlation of WGCNA modules with different clinical traits.** Heatmap depicting correlation values calculated between a variety of patient clinical traits and modules identified by their WGCNA eigengene vectors. Modules are represented by the rows of this matrix and have been named according to their most significant correlated trait and/or a pathway enrichment analysis. Trees are illustrating the outcome of hierarchical clustering performed on the data.


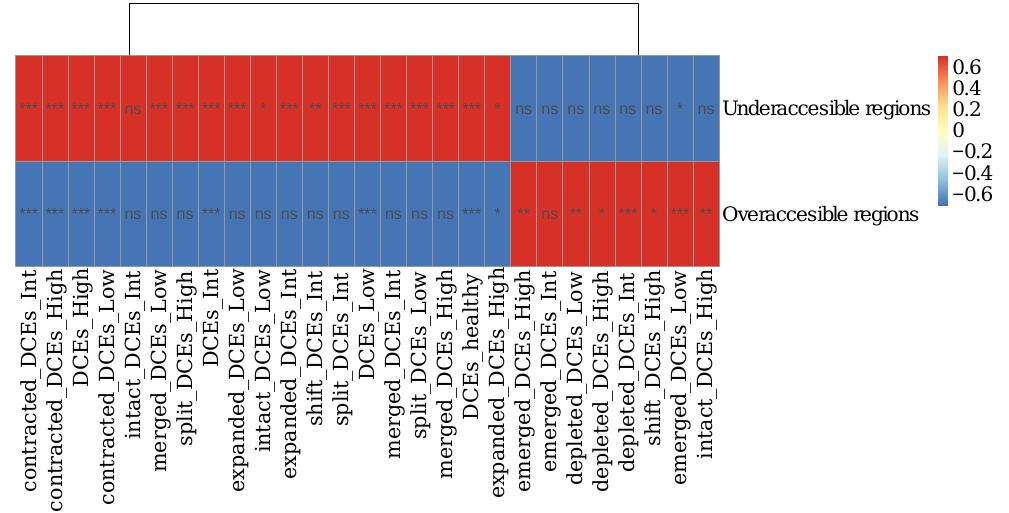


**Supplementary Figure 7. Enrichment of DCEs in differentially accessible regions.** Heatmap illustrating the results of an enrichment test for DCEs in differentially accessible regions. Scaling and centering has been performed per column. All the different DCE categories have been tested. Symbols inside cells demonstrate the significance level of the outcome of each test (*:0.05; **:0.01; ***:0.001). Significance has been assessed by a non-parametric, permutation-based test.

**
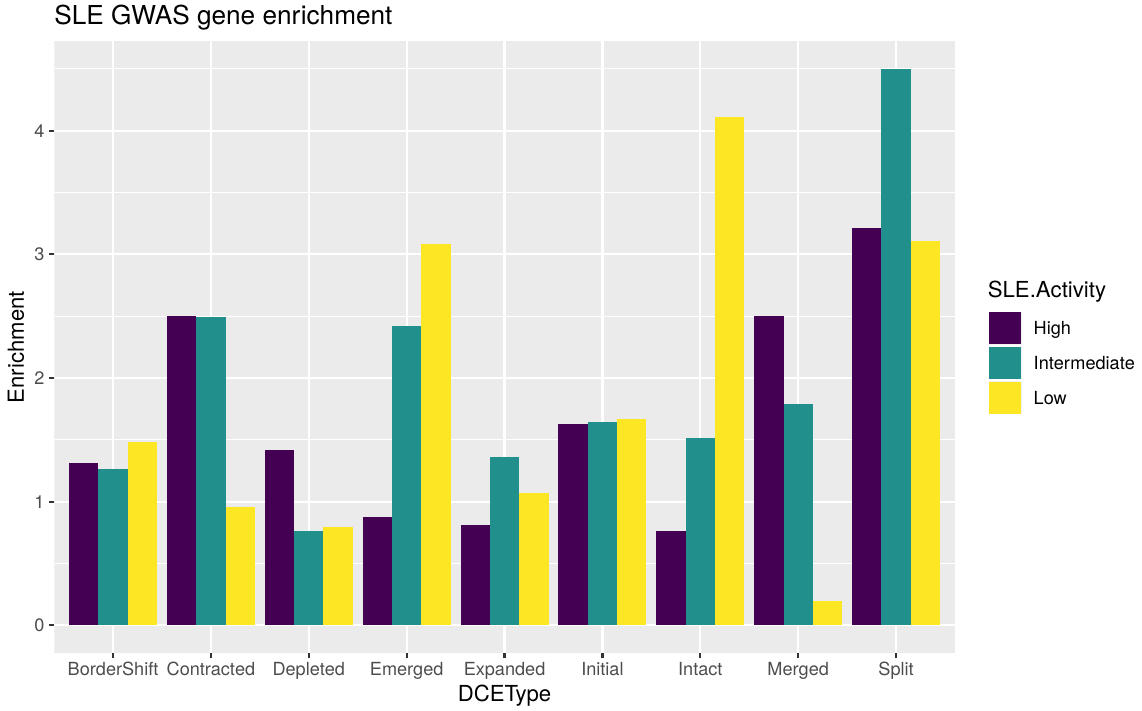
**

**Supplementary Figure 8. Enrichment of SLE genetically associated genes among different DCE types.** Bars correspond to fold-enrichment of overlap between a set of genes reported as genetically associated with SLE (downloaded from GWAS Central, December 2019) against different types of DCE for each of the three disease activity groups. All values >2 were significant at p-value<0.01.

**References**

1. Panousis, N. I. *et al.* Combined genetic and transcriptome analysis of patients with SLE: distinct, targetable signatures for susceptibility and severity. *Ann. Rheum. Dis.* annrheumdis-2018-214379 (2019) doi:10.1136/annrheumdis-2018-214379.

2. Zaborowski, R. & Wilczyński, B. BPscore: An Effective Metric for Meaningful Comparisons of Structural Chromosome Segmentations. *J. Comput. Biol.* **26**, 305–314 (2019).

3. Zyla, J. *et al.* Gene set enrichment for reproducible science: comparison of CERNO and eight other algorithms. *Bioinformatics* (2019) doi:10.1093/bioinformatics/btz447.
